# A multicenter cross-sectional study on proactive health knowledge, attitude, and practice and its influencing factors among resident standardized training physicians in Guangxi

**DOI:** 10.3389/fpubh.2026.1886672

**Published:** 2026-08-28

**Authors:** Qian Zeng, Jichen He, Kun Ye, Xuepeng Du, Jianzhen Chang, Qiaoying Wei, Shenglin Liang

**Affiliations:** The People’s Hospital of Guangxi Zhuang Autonomous Region and Guangxi Academy of Medical Sciences, Nanning, Guangxi, China

**Keywords:** cross-sectional study, influencing factors, knowledge-attitude-practice, proactive health, resident standardized training physicians

## Abstract

**Objective:**

The purpose of this study is to examine the state of proactive health knowledge, attitude, and practice (KAP) among Guangxi’s resident standardized training physicians as well as the factors that affect it. The results will be used to optimize health management strategies.

**Methods:**

A multicenter cross-sectional survey was conducted. Univariate analysis was used to compare differences in proactive health KAP among residents with different characteristics, and multivariate linear regression was performed to identify associated factors.

**Results:**

A total of 1,214 residents were included. The overall mean scores of proactive health knowledge, attitude, and practice were 4.32 ± 0.72, 4.40 ± 0.69, and 3.89 ± 0.64, respectively. Gender, household registration type, educational level, training identity, and region were significantly associated with KAP scores (all *p* < 0.05). Males had lower knowledge (*β* = −0.239, *p* < 0.001) and attitude (*β* = −0.183, *p* < 0.001) scores but higher practice scores (*β* = 0.085, *p* < 0.05) than females. Residents with agricultural household registration had lower knowledge (*β* = −0.124, *p* < 0.05) and attitude (*β* = −0.126, *p* < 0.05) scores. Physicians from western and northern Guangxi had remarkably lower knowledge and practice scores (*p* < 0.05); additionally, residents in northern Guangxi also obtained significantly poorer attitude scores (*β* = −0.146, *p* < 0.05). Practice scores were higher in residents who were staff of the training base hospital (*β* = 0.307, *p* < 0.05).

**Conclusion:**

Standardized residents in Guangxi generally have sound proactive health knowledge and positive health attitudes. However, their practice score is markedly lower than the knowledge score and attitude score, which creates an obvious KAP gap. Obvious intergroup disparities exist in residents’ KAP levels. Targeted hierarchical health education should be carried out, with special attention paid to male residents, physicians with agricultural household registration, and practitioners in underdeveloped western and northern Guangxi, so as to bridge the gap between health cognition, positive attitudes and healthy behaviors.

## 1 Introduction

China’s standardized residency training system was formally established in late 2013. Standardized residency training serves as the cornerstone of postgraduate medical education in China and represents a critical transition for clinical physicians from academic training to independent practice (1). In recent years, China has continuously improved the construction of the residency training system. Through policies such as the integration of medical education and clinical practice, standardized management of training bases, prioritization of shortage specialties, and guaranteed benefits, the system has expanded to cover key fields such as general practice, pediatrics, and critical care medicine, gradually achieving balanced development in training quality and talent supply (2, 3). At the national level, policy coordination between residency training and professional title advancement, job appointments, and academic degree pathways has been continuously strengthened. The “Two Equivalences” policy has been clarified to safeguard the career development rights of residents, thereby supplying the healthcare system with standardized, high-quality clinical backbone personnel (4–6). However, the combination of high-intensity clinical rotations, frequent night shifts, academic pressure, and heavy workloads has made residents a high-risk group in terms of physical and mental health. Their health literacy, health beliefs, and health behaviors not only affect their own career sustainability but also directly impact the quality of clinical care and patient safety (7, 8). Currently, research on proactive health among resident physicians in China remains limited, and the lack of regional empirical data hinders the development of targeted health interventions and career support strategies.

Proactive health is a health management model centered on the individual, focused on prevention, and spanning the entire life cycle. It emphasizes the proactive acquisition of health knowledge, the establishment of positive health beliefs, and the practice of healthy behaviors, while maintaining health through self-monitoring and early intervention. This aligns with the strategic direction of “Healthy China 2030” to shift from a “disease-centered” approach to a “people’s health-centered” approach. Current research on proactive health focuses on multiple populations, employing methods such as cross-sectional studies, the Delphi method, and questionnaires to explore behavioral status, influencing factors, and management models. Study subjects include community-based older patients with hypertension, community general practitioners, and healthcare workers while also covering chronic disease management practices in large public hospitals. Research findings indicate that studies on the current status of proactive health behaviors among community-based older patients with hypertension remain limited, necessitating the identification of influencing factors to optimize interventions. Community general practitioners demonstrate positive beliefs regarding proactive health management but face implementation barriers-such as insufficient policy support-due to gaps in systematic knowledge and behavioral implementation, which are influenced by factors like educational background and professional titles (9). Large public hospitals, by establishing multidimensional proactive health service models, provide practical insights for integrating medical treatment and prevention in chronic disease management (10). Internationally, proactive health has established mature theoretical frameworks such as the Knowledge-Attitude-Belief-Practice (KABP) model, the Health Belief Model (HBM), and the Theory of Planned Behavior (TPB), which are widely applied in health promotion and chronic disease prevention and control among working populations (11–13). Research in China is gradually deepening in areas such as the definition of proactive health, the empowerment of digital health technologies, and the enhancement of health literacy across the entire population. Studies focusing on healthcare workers have primarily targeted practicing physicians and nurses, revealing prominent issues such as occupational stress, sleep disorders, and unhealthy lifestyles; however, there has been no systematic focus on the specific transitional group of resident physicians (14).

Proposed by Ramsey and Rickson in 1976, the KAP model serves as a classic analytical framework to interpret the transformation pathway of individual cognition and behaviors in health education or behavioral interventions (15). Bloom’s taxonomy of educational objectives has been empirically verified in standardized physician training, which establishes a hierarchical teaching system covering cognition, affection and skills to facilitate the translation of theoretical knowledge into clinical behaviors (16). The KAP-based research conducted by Liu et al. (17) further confirms that attitude acts as a critical mediator between knowledge and practice, which is highly consistent with the affective domain of Bloom’s theory. These two theories complement each other: Bloom’s taxonomy guides the design of hierarchical teaching interventions, while the KAP model quantifies corresponding changes in knowledge, attitudes and behaviors. Collectively, they construct a complete theoretical pathway for the present study to evaluate the training outcomes of proactive health competency via the KAP model.

There is an irreplaceable necessity to study resident physicians. First, resident physicians are in a critical phase of professional development, and their health perceptions and behavioral patterns will have a long-term impact on their health status and professional longevity throughout their careers. Second, as health role models and educators, resident physicians’ own levels of proactive health directly influence the effectiveness of patient health guidance and the quality of doctor-patient communication, making them a vital link in enhancing the health literacy of the entire population (18). Furthermore, Guangxi has seen a continuous expansion in the scale of its residency training programs. However, the region’s economic conditions, healthcare resources, and occupational environment may introduce unique health-related factors, creating an urgent need for locally relevant data (19, 20). Finally, existing studies often focus on mental health or single health behaviors, lacking a comprehensive assessment of proactive health knowledge, attitude, and practice (KAP), as well as an analysis of the underlying mechanisms of these factors, which hinders the design of targeted intervention strategies (21, 22).

This study targets resident physicians in Guangxi and aims to systematically assess their current levels of proactive health knowledge, health beliefs, and health behavior practices. It seeks to identify key influencing factors-including demographic characteristics, training environments, occupational stress, and social support-and construct a regional model of factors influencing proactive health among resident physicians. By clarifying current shortcomings and underlying mechanisms, this study provides empirical evidence for developing targeted health promotion strategies, optimizing health management services during residency training, and enhancing the physical and mental well-being as well as the professional competence of resident physicians. Additionally, it serves as a reference for similar research and intervention practices in other regions of China, contributing to the improvement of a comprehensive health protection system for medical professionals throughout their career lifecycle and promoting the high-quality development of the residency training system.

## 2 Methods

### 2.1 Study participants

This cross-sectional study was carried out from January 2 to 15, 2025 in Guangxi Zhuang Autonomous Region, China. Convenience sampling was adopted. Under the assistance of administrators from each training base, residents receiving standardized training completed questionnaires through the online platform Wenjuanxing. The data collection method used in this study was consistent with that reported in previous literature, and the detailed sampling procedure is described in the corresponding reference (23).

Prior to data collection, all eligible participants were fully informed of the study objectives, questionnaire filling procedures, data usage rules, and privacy protection protocols. Participants were clearly notified that participation was completely voluntary; they could withdraw from the survey at any time without any adverse impact on their standardized residency training assessments. Each participant signed a electronic informed consent form before accessing the questionnaire system via a shared QR code. A series of anonymization measures were adopted throughout the survey to protect personal privacy: no personally identifiable information, including full names, ID numbers, or training departments, was collected. The platform automatically generated an independent anonymous unique identifier for each valid response, which was not linked to any personal identity data. To prevent duplicate submissions, each IP address and electronic device was limited to one submission. The system only automatically recorded questionnaire completion time and geographic location for response validity verification. All location data was encrypted and stored separately from questionnaire responses, and permanent de-identification was performed upon completion of data collection (24).

The inclusion criteria for participants in this study were as follows: (1) age over 18; (2) currently participating in standardized residency training in Guangxi; (3) willing to participate in the survey; (4) not yet having completed the training program. Exclusion criteria: (1) those unable to access the questionnaire system or complete the questionnaire during the survey period due to withdrawal from training, deferral of training, or leave of absence; (2) participants who voluntarily exited the survey during the questionnaire-filling process; (3) participants with incomplete questionnaires (missing key information) or logical errors (e.g., contradictory scores for different items within the same dimension); (4) participants who took longer than 30 min to complete the questionnaire. This study has been approved by the Ethics Committee of the People’s Hospital of Guangxi Zhuang Autonomous Region (Approval No.: KY-KJT-2023-206). All survey procedures were performed in strict accordance with ethical requirements for human subject research.

### 2.2 Development of the proactive health KAP scale

This study developed a Proactive Health Knowledge–Attitude–Practice (KAP) scale tailored for standardized resident physicians by integrating literature review, expert consultation, and pilot testing. Guided by the KAP theoretical framework, we systematically reviewed literature on proactive health, referenced China’s national health service surveys and the Chinese Dietary Guidelines (2022), and incorporated occupational characteristics of resident physicians to preliminarily generate 36 initial items, including 13 knowledge items, 11 attitude items, and 12 practice items.

Two rounds of offline expert panel reviews were conducted with eight experts who held associate senior or higher professional titles and possessed rich experience in standardized residency training teaching or administration, including clinical preceptors and full-time residency management staff. The first round of expert discussion identified four core dimensions of proactive health: health responsibility, physical exercise, nutritional diet, and healthy behaviors. Experts recommended consistent item structures covering knowledge, attitude, and practice within each dimension to improve systematicness and structural coherence. The research team revised the initial items accordingly, adjusting their dimensional assignments and revising redundant, ambiguous, and misclassified items. The second round of expert consultation further refined all items in terms of scientific rigor, representativeness, readability, and suitability for resident physicians. The finalized standardized scale contained four dimensions, each with four knowledge items, four attitude items, and four practice items, yielding a total of 48 items.

A pilot test was performed among 73 standardized resident physicians at Liuzhou Workers’ Hospital to evaluate item comprehensibility, completion time, and overall acceptability. All participants finished the questionnaire independently without proposing any revisions, so all 48 items were retained for the formal survey. Reliability and validity analyses were performed on formal survey data. The scale yielded a Cronbach’s *α* coefficient of 0.866 and a Kaiser-Meyer-Olkin (KMO) value of 0.836, indicating satisfactory internal consistency and construct validity.

### 2.3 Survey instrument

The research team independently designed the “Survey Questionnaire on the Current Status of Knowledge, Attitude, and Practice Regarding Proactive Health Among Resident Physicians in Guangxi.” The questionnaire consists of two sections: the first covers basic information, including age, gender, household registration type, ethnicity, marital status, and educational attainment; the second comprises a scale assessing resident physicians’ knowledge, attitude, and practice regarding proactive health, which includes four dimensions-health responsibility, physical exercise, nutritional diet, and health behaviors-with four items in each dimension. The proactive health KAP scale adopted a 5-point Likert scoring system. Items were scored from “strongly disagree (1 point)” to “strongly agree (5 points)” or “never (1 point)” to “always (5 points)” according to the setting of different dimensions. The score of each dimension was the sum of the scores of its corresponding items, and the total scale score was the sum of all dimension scores. Higher scores indicated higher levels of proactive health knowledge, attitudes and practices among resident physicians under standardized training (25). The Cronbach’s alpha coefficient for the KAP Scale was 0.866, the Kaiser–Meyer–Olkin (KMO) measure of sampling adequacy was 0.836, and the Bartlett’s sphericity test value was 779.362 (*p* < 0.001), indicating good sampling adequacy. Five factors with eigenvalues greater than 1 were extracted, explaining 57.46% of the total variance, with factor loadings ranging from 0.576 to 0.840. This indicates that the questionnaire demonstrates good consistency in both reliability and validity, supporting its use in this study to assess resident physicians’ mastery of proactive health knowledge, levels of health attitude, and current health behavior practices.

### 2.4 Covariates

This study collected basic sociodemographic data from resident physicians at medical institutions and adjusted for these factors as covariates. The covariates included age, gender (male/female), ethnicity (Han Chinese/ethnic minorities), marital status (unmarried/married), household registration type (non-agricultural/agricultural), and region (Eastern Guangxi/Southern Guangxi/Western Guangxi/Northern Guangxi/Central Guangxi). Social factors included year of training (first year/s year/third year), residency status (integrated professional master’s degree graduate student/trainee from other institutions/self-funded trainee/staff of the training base hospital), educational level (bachelor’s degree or below/currently pursuing a professional master’s degree/master’s degree or above), recent graduate (yes/no), passing the Physician Qualification Examination (passed/failed), and major category (Emergency and Critical Care Medicine/Dentistry/Obstetrics and Gynecology and Pediatrics/Medical Technology/General Practice/Internal Medicine/Surgery).

### 2.5 Statistical analysis

Data cleaning and statistical analysis were performed using SPSS 25.0 and Stata 18.0. Descriptive statistics were performed via SPSS, while univariate and multivariate analyses were conducted using Stata. Continuous variables that followed a normal distribution were described as mean ± standard deviation, while categorical variables were described as frequency (%). Differences among survey participants with different sociodemographic characteristics were analyzed using the two-sample *t*-test or one-way analysis of variance (ANOVA) (26). A multiple linear regression model was employed to analyze the factors influencing KAP. The significance level was set at *α* = 0.05 (two-tailed).

## 3 Results

### 3.1 Basic sociodemographic characteristics

This study included a total of 1,214 resident physicians, with a mean age of (25.46 ± 1.98) years. Regarding sociodemographic characteristics, 461 were male (37.97%), and 753 were female (62.03%); 808 had rural household registration (66.56%); 820 were of Han ethnicity (67.55%); 1,152 (94.89%) were unmarried, divorced, or widowed; 910 (74.96%) had an educational attainment of a bachelor’s degree or lower. There were 534 first-year residents (43.99%), 630 social trainees (51.89%), 958 non-recent graduates (78.91%), and 806 individuals who had passed the Physician Qualification Examination (60.60%). In terms of specialty distribution, Internal Medicine had the highest proportion, with 329 participants (27.1%). Geographically, 384 participants (31.63%) were from the Central Guangxi region. Detailed demographic and occupational characteristics are presented in Table 1.

**Table 1:** Baseline sociodemographic and occupational characteristics of study participants (*n* = 1,214).

Variable	*n*	***%***
Age, years, mean ± SD	25.46 ± 1.98	
Gender		
Female	753	62.03
Male	461	37.97
Household registration		
Non-agricultural	406	33.44
Agricultural	808	66.56
Ethnicity		
Han	820	67.55
Minority	394	32.45
Marital status		
Unmarried	1,152	94.89
Married	62	5.11
Educational level		
Bachelor or below	910	74.96
Master in-service	199	16.39
Master or above	105	8.65
Standardized training grade		
Grade 1	534	43.99
Grade 2	368	30.31
Grade 3	312	25.70
Training identity		
Master integrated	254	20.92
Other institution-sponsored	301	24.79
Social recruit	630	51.89
Training base staff	29	2.39
Fresh graduate?		
Yes	256	21.09
No	958	78.91
Passed licensed physician qualification exam?		
Yes	734	60.46
No	480	39.54
Major category		
Emergency and critical care medicine	41	3.38
Stomatology department	48	3.95
Obstetrics, gynecology and pediatrics	107	8.81
Medical technology	142	11.7
General practice	240	19.77
Internal medicine	329	27.1
Surgery	307	25.29
Region of Guangxi		
Eastern	255	21.00
Southern	197	16.23
Western	168	13.84
Northern	210	17.30
Central	384	31.63
Total	1,214	100.00

Data are presented as *n* (%) for categorical variables and mean ± SD for continuous variables.

### 3.2 Current status of proactive health KAP

#### 3.2.1 Knowledge

**Figure 1:**
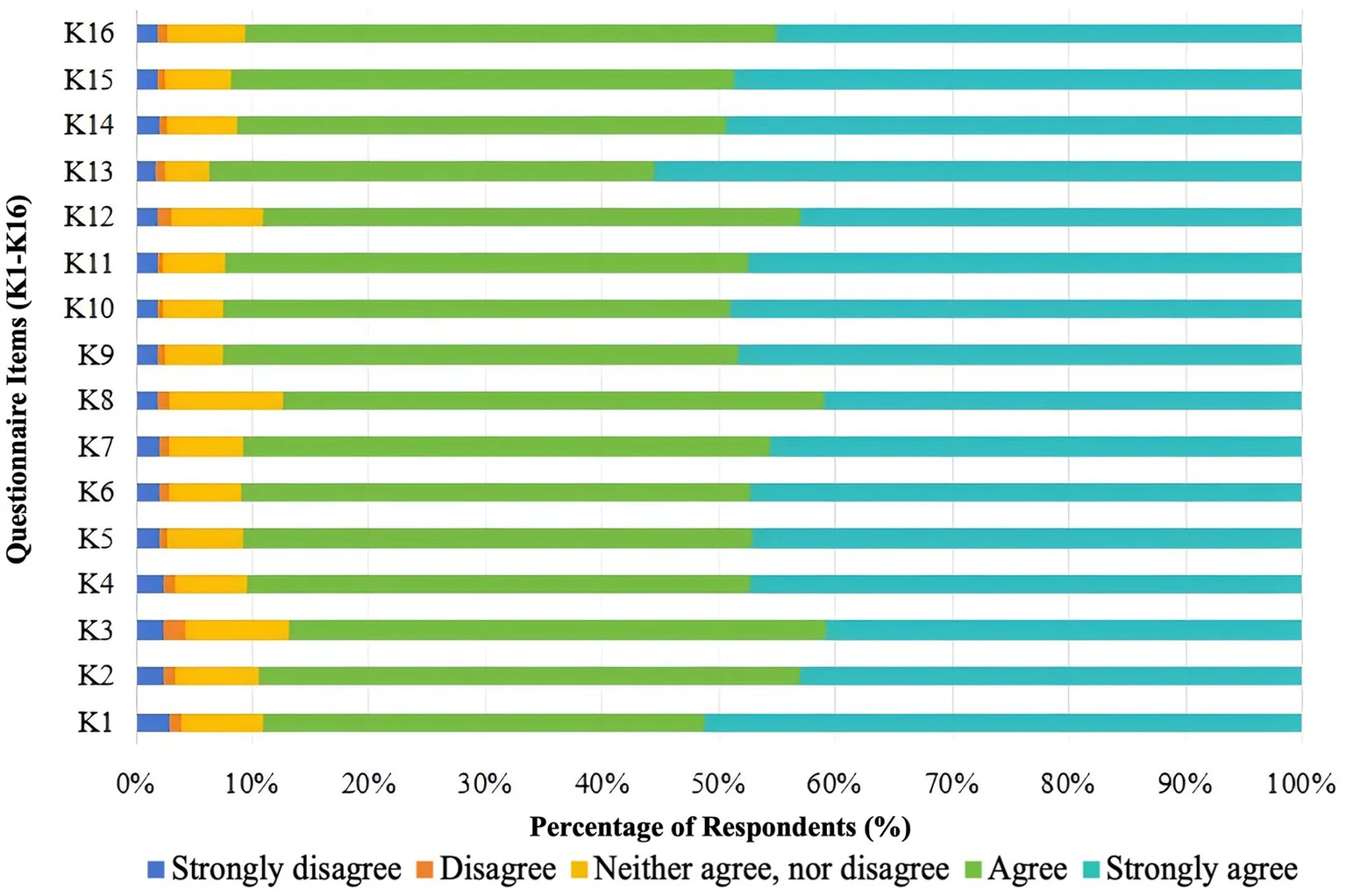
Level of proactive health knowledge among resident standardized training physicians in Guangxi. (1) K1: I understand the importance of health checkups and know that regular checkups help identify potential health risks; K2: I know that when I feel unwell, I can relieve symptoms through medication or self-care measures (such as rest and dietary adjustments); K3: I know how to access health support through online platforms (such as websites, apps, and social media accounts); K4: I understand the importance of following medical advice and actively pursuing treatment after a diagnosis; K5: I understand the health benefits of light exercise (such as walking or calisthenics); K6: I know that moderate exercise (such as playing volleyball, jogging, or practicing Tai Chi) can effectively improve physical health; K7: I know the importance of moderate-intensity exercise (such as cycling or running) for health management; K8: I know how high-intensity exercise (such as badminton and basketball) helps improve health; K9: I understand the health benefits of eating fresh vegetables and fruits daily; K10: I know that consuming appropriate amounts of fish, poultry, eggs, and other foods is beneficial to physical health; K11: I understand the role of a light diet in controlling weight and reducing the risk of chronic diseases; K12: I know how to plan three balanced meals a day and ensure adequate water intake; K13: I understand the health risks of smoking and recognize the importance of quitting; K14: I know that getting at least 7 h of sleep per day has a positive impact on health; K15: I know how to moderate alcohol consumption to maintain good health; K16: I understand the negative health effects of excessive smartphone or computer use.

In the knowledge index, it was found that over 90% of resident physicians agree on the importance of understanding and mastering proactive health knowledge. In addition, 93.66% of residents agreed that smoking is harmful to health and recognized the importance of quitting; 92.50% of residents were aware that consuming appropriate amounts of fish, poultry, eggs, and other foods is beneficial to physical health; 91.35% of residents knew that getting at least 7 h of sleep per day has a positive impact on health; 89.37% of residents were aware that when feeling unwell, symptoms can be alleviated through medication or self-management measures (such as rest and dietary adjustments), as shown in Figure 1. The average score for this index was 4.32 ± 0.72. There were statistically significant differences in average scores across gender, household registration type, educational level, residency status, and region (*p* < 0.05), as shown in Table 2 and Figure 1.

**Table 2:** Comparison of proactive health KAP scores by sociodemographic and occupational characteristics.

Variable	Knowledge				Attitude				Practice			
	Mean	Std	*t*/*F*	*p*	Mean	Std	*t*/*F*	*p*	Mean	Std	*t*/*F*	*p*
Total	4.32	0.72			4.40	0.69			3.89	0.64		
Gender			4.332	<0.001			3.278	0.001			−2.939	0.003
Female	4.39	0.61			4.45	0.60			3.85	0.60		
Male	4.21	0.87			4.31	0.81			3.96	0.70		
Household registration			2.149	0.031			2.381	0.017			0.956	0.340
Non-agricultural	4.39	0.71			4.46	0.67			3.91	0.65		
Agricultural	4.29	0.73			4.36	0.70			3.88	0.64		
Ethnicity			1.596	0.110			1.226	0.220			0.841	0.400
Han	4.35	0.71			4.41	0.69			3.90	0.64		
Minority	4.28	0.74			4.36	0.70			3.87	0.65		
Marital status			−0.117	0.906			0.314	0.753			−1.530	0.126
Unmarried	4.32	0.72			4.40	0.68			3.88	0.63		
Married	4.33	0.86			4.37	0.86			4.01	0.77		
Educational level			3.230	0.040			1.660	0.190			5.880	0.003
Bachelor or below	4.34	0.71			4.41	0.69			3.92	0.63		
Master in-service	4.21	0.82			4.31	0.73			3.75	0.67		
Master or above	4.37	0.60			4.42	0.61			3.91	0.63		
Standardized training grade			2.227	0.328			0.980	0.612			3.544	0.170
Grade 1	4.34	0.71			4.41	0.67			3.92	0.61		
Grade 2	4.34	0.75			4.40	0.71			3.88	0.70		
Grade 3	4.29	0.73			4.36	0.71			3.84	0.62		
Training identity			4.390	0.004			2.890	0.034			8.280	<0.001
Master integrated	4.19	0.85			4.29	0.79			3.73	0.70		
Other institution-sponsored	4.32	0.65			4.39	0.63			3.88	0.58		
Social recruit	4.37	0.69			4.44	0.67			3.95	0.64		
Training base staff	4.47	0.78			4.48	0.80			4.12	0.53		
Fresh graduate?			−0.139	0.890			−0.080	0.936			1.456	0.146
Yes	4.32	0.88			4.39	0.83			3.94	0.69		
No	4.33	0.68			4.40	0.65			3.88	0.63		
Passed licensed physician qualification exam?			−0.320	0.749			−0.892	0.373			−1.089	0.276
Yes	4.32	0.74			4.38	0.72			3.87	0.66		
No	4.33	0.70			4.42	0.65			3.91	0.61		
Major category			5.359	0.499			7.539	0.274			5.130	0.527
Emergency and critical care medicine	4.37	0.54			4.51	0.54			3.93	0.60		
Stomatology department	4.29	0.65			4.29	0.60			3.92	0.60		
Obstetrics, gynecology and pediatrics	4.41	0.51			4.47	0.49			3.83	0.62		
Medical technology	4.29	0.77			4.36	0.75			3.91	0.66		
General practice	4.30	0.65			4.37	0.63			3.85	0.57		
Internal medicine	4.36	0.78			4.43	0.73			3.92	0.65		
Surgery	4.29	0.79			4.37	0.76			3.88	0.70		
Region of Guangxi			7.170	<0.001			4.500	0.001			9.110	<0.001
Eastern	4.40	0.63			4.45	0.63			3.98	0.57		
Southern	4.49	0.59			4.55	0.56			4.09	0.56		
Western	4.13	0.87			4.30	0.73			3.79	0.61		
Northern	4.24	0.77			4.30	0.75			3.81	0.72		
Central	4.32	0.73			4.38	0.73			3.82	0.67		

Data are presented as mean ± SD. Independent-samples *t*-test or one-way ANOVA was used for group comparisons. *p* < 0.05 was considered statistically significant. The “training base staff” subgroup has a small sample size (*n* = 29), and the conclusion of the highest practice score has limited robustness.

#### 3.2.2 Attitude

**Figure 2:**
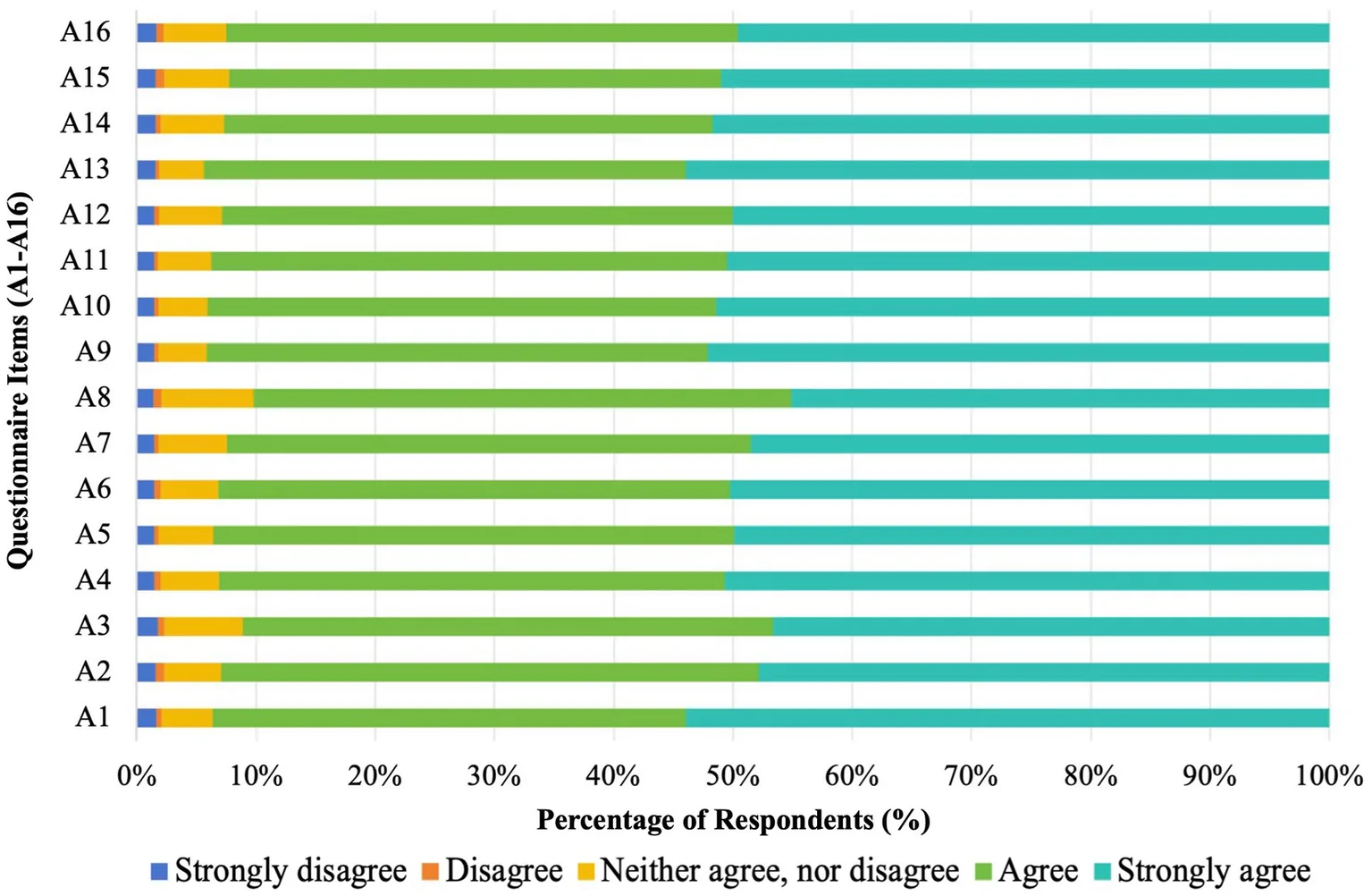
Level of proactive health attitude among resident standardized training physicians in Guangxi. A1: I believe that regular health checkups are very important for detecting health issues and preventing disease; A2: I believe that self-medication or self-management measures (such as taking medication or resting) are effective ways to alleviate physical discomfort; A3: I believe that actively seeking health information and support through health platforms (such as websites, apps, and social media accounts) is helpful for my health management; A4: I believe that following a doctor’s instructions and undergoing standardized treatment are effective ways to manage disease and improve health; A5: I believe engaging in light exercise (such as walking or calisthenics) is a necessary measure for improving health; A6: I believe moderate exercise (such as playing volleyball or jogging) is very important for maintaining health; A7: I believe engaging in moderate-intensity exercise (such as running or cycling) is crucial for physical health; A8: I believe high-intensity exercise (such as badminton or basketball) helps improve physical health; A9: I believe eating fresh vegetables and fruits every day is very beneficial to my health; A10: I believe consuming appropriate amounts of fish, poultry, eggs, and other foods is key to maintaining a healthy diet; A11: I believe maintaining a light diet can help control weight and prevent health problems; A12: I believe eating three balanced meals a day and drinking enough water are important factors in maintaining health; A13: I believe quitting smoking can effectively reduce health risks and improve quality of life; A14: I believe getting enough sleep every day (at least 7 h) is very important for health; A15: I believe moderating alcohol intake is a necessary measure for maintaining good health; A16: I believe reducing screen time helps prevent health issues (such as eye strain and insomnia).

In the attitude index, it was found that over 90% of resident physicians agreed on the importance of taking proactive health measures to maintain good health. In addition, 94.40% of resident physicians believed that quitting smoking can effectively reduce health risks and improve quality of life, while 91.10% agreed that actively seeking health information and support through health platforms (such as websites, apps, and WeChat official accounts) is helpful for their health management, as shown in Figure 2. The average score for this index was 4.40 ± 0.69. There were statistically significant differences in average scores across gender, household registration type, residency status, and region (*p* < 0.05), as shown in Table 2 and Figure 2.

#### 3.2.3 Practice

**Figure 3:**
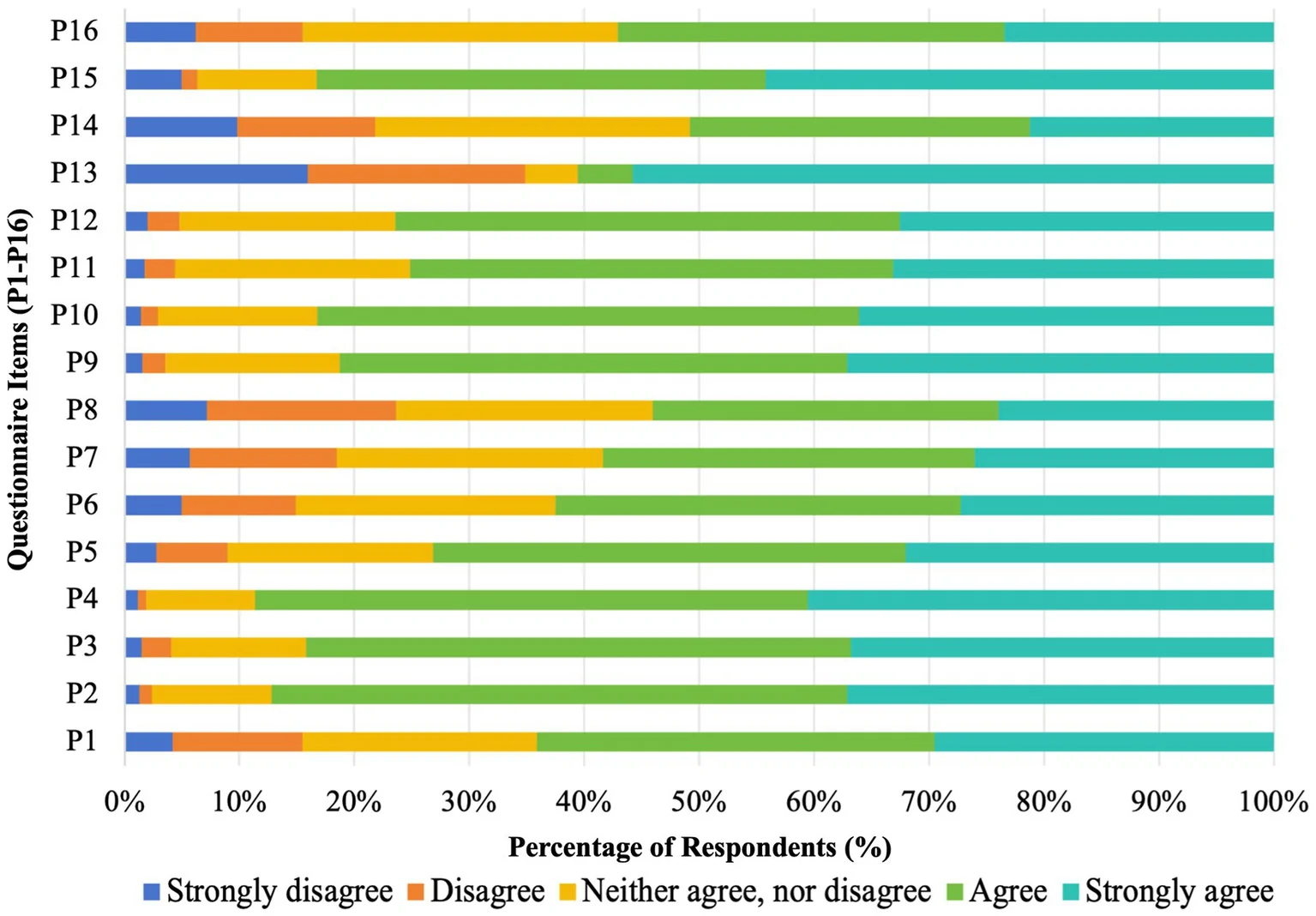
Level of proactive health practice among resident standardized training physicians in Guangxi. (1) P1: You undergo a health checkup every year; P2: When you feel unwell, you proactively take medication or self-care measures (such as rest or dietary adjustments); P3: When you feel unwell, you proactively seek health advice through health platforms (such as websites or apps); P4: After a health issue is diagnosed, you follow medical advice and actively undergo proper treatment; P5: I engage in light exercise (such as walking or calisthenics) every week; P6: I engage in moderate-intensity exercise (such as volleyball, jogging, or tai chi) every week; P7: I engage in moderate-intensity exercise (such as running or cycling) every week; P8: I engage in high-intensity exercise (such as badminton or basketball) every week; P9: I eat fresh vegetables and fruits every day; P10: I consume appropriate amounts of fish, poultry, eggs, and lean meat; P11: I maintain a relatively light diet and avoid excessive amounts of greasy foods; P12: I eat three balanced meals a day, ensure adequate hydration, and drink water at appropriate intervals; P13: I smoked every day during the past month; P14: I ensured I got at least 7 h of sleep every day during the past month; P15: I control my alcohol intake to maintain a healthy level; P16: I often feel fatigued and drowsy due to excessive use of my smartphone or computer.

The practice index revealed that 88.63% of resident physicians follow medical advice and actively undergo standardized treatment after being diagnosed with a health issue. Additionally, 87.23% of resident physicians take medication or self-care measures (such as rest or dietary adjustments) when they feel unwell. Furthermore, in the past month, 60.58% of residents smoked daily, while 62.52% engaged in moderate-intensity exercise weekly (such as playing volleyball, jogging, or practicing tai chi). Moreover, 50.82% of residents ensured they slept at least 7 h daily in the past month, as shown in Figure 3. Regarding overall practice index performance, the average score was 3.89 ± 0.64, with statistically significant differences in average scores across gender, educational level, residency status, and region (*p* < 0.05), as shown in Table 2 and Figure 3.

### 3.3 Comparison of KAP scores by different characteristics

In this study, the overall mean scores for KAP were 4.32 ± 0.72, 4.40 ± 0.69, and 3.89 ± 0.64, respectively. Analysis and comparison of the average scores for proactive health KAP among resident physicians revealed that gender, household registration type, educational level, residency status, and region significantly influenced the average scores for KAP (*p* < 0.05), as shown in Table 2.

With regard to gender, there were significant differences between men and women in scores for knowledge (*p* < 0.001), beliefs (*p* = 0.001), and behaviors (*p* = 0.003). Women had higher average scores than men for proactive health knowledge (4.39 ± 0.61) and beliefs (4.45 ± 0.60), but their average behavior score (3.85 ± 0.60) was lower than that of men; Regarding household registration type, there were statistically significant differences in the mean knowledge and belief scores between physicians with agricultural and non-agricultural household registrations (*p* < 0.05), whereas the differences in mean behavior scores were not statistically significant (*p* > 0.05). Physicians with non-agricultural household registrations had higher mean knowledge scores (4.39 ± 0.71) and belief scores (4.46 ± 0.67) than those with agricultural household registrations; Regarding educational attainment, there were statistically significant differences in the mean scores for knowledge and behavior among physicians with different educational levels (p < 0.05), while there were no statistically significant differences in the mean scores for beliefs (*p* > 0.05). Physicians with a master’s degree or higher had the highest average knowledge score (4.37 ± 0.60), while those with a bachelor’s degree or lower had the highest average behavioral score (3.92 ± 0.63); physicians currently enrolled in professional master’s programs had the lowest average scores for both knowledge and behavior; Regarding residency status, there were significant differences in the average scores for knowledge (*p* = 0.004), beliefs (*p* = 0.034), and behavior (*p* < 0.001) among physicians with different statuses. Physicians employed by the training base hospital had the highest average scores for knowledge (4.47 ± 0.78), beliefs (4.48 ± 0.80), and behaviors (4.12 ± 0.53) were the highest; physicians with the residency training status of integrated professional master’s students had the lowest average scores for knowledge (4.19 ± 0.85), beliefs (4.29 ± 0.79), and behaviors (3.73 ± 0.70); In terms of region, there were significant differences in the mean scores for knowledge (*p* < 0.001), beliefs (*p* = 0.001), and behaviors (*p* < 0.001) among physicians from different regions. Physicians in the Southern Guangxi region had the highest mean scores for knowledge (4.49 ± 0.59), beliefs (4.55 ± 0.56), and behaviors (4.09 ± 0.56), while the Western Guangxi region had the lowest average scores for knowledge (4.13 ± 0.87), beliefs (4.30 ± 0.73), and behaviors (3.79 ± 0.61). However, there were no significant differences in average scores for KAP based on ethnicity, marital status, year of study, whether the physician was a recent graduate, whether they had passed the Physician Qualification Examination, or specialty classification.

### 3.4 Multivariate linear regression analysis

The results of multiple linear regression analysis show that, after controlling for confounding factors, men scored lower than women on proactive health knowledge (*β* = −0.239, *p* < 0.001) and beliefs (*β* = −0.183, *p* < 0.001); however, men scored higher on proactive health behaviors (*β* = 0.085, *p* < 0.05). When analyzed by household registration type, physicians with agricultural household registration scored lower on knowledge (*β* = −0.124, *p* < 0.05) and beliefs (*β* = −0.126, *p* < 0.05). Physicians from western and northern Guangxi demonstrated markedly lower knowledge and practice scores (*p* < 0.05); additionally, residents in northern Guangxi also showed significantly poorer attitude scores (*β* = −0.146, *p* < 0.05). Those whose residency training was conducted at the training base hospital scored higher on behavioral measures (*β* = 0.307, *p* < 0.05). This indicates that knowledge scores were significantly associated with being male, having an agricultural household registration, and being from the western and northern regions of Guangxi; belief scores were significantly associated with being male, having an agricultural household registration, and being from the northern region of Guangxi; and behavioral scores were significantly associated with being male, region, and having residency training conducted at the training base hospital, as shown in Table 3.

**Table 3:** Multivariate linear regression analysis of factors associated with proactive health KAP.

Variable	Knowledge					Attitude					Practice				
	*β*	*p*	95%CI		VIF	*β*	*p*	95%CI		VIF	*β*	*p*	95%CI		VIF
			Lower	Upper				Lower	Upper				Lower	Upper	
Age	0.011	0.463	−0.019	0.042	2.24	0.007	0.623	−0.022	0.036	2.24	0.034	0.014	0.007	0.061	2.24
Gender (reference: female)					1.19					1.19					1.19
Male	−0.239	<0.001	−0.329	−0.149		−0.183	<0.001	−0.270	−0.097		0.085	0.036	0.006	0.165	
Household registration type (reference: non-agricultural)					1.07					1.07					1.07
Agricultural	−0.124	0.005	−0.212	−0.037		−0.126	0.003	−0.210	−0.042		−0.030	0.451	−0.108	0.048	
Ethnicity (reference: Han nationality)					1.13					1.13					1.13
Ethnic minority	−0.036	0.440	−0.126	0.055		−0.033	0.459	−0.120	0.054		0.004	0.916	−0.076	0.085	
Marital status (reference: married)					1.28					1.28					1.28
Unmarried	0.001	0.990	−0.204	0.207		−0.041	0.683	−0.239	0.157		0.020	0.830	−0.163	0.203	
Education level (reference: bachelor and below)															
Master in-service	0.072	0.475	−0.126	0.269	3.34	0.087	0.372	−0.104	0.277	3.34	0.011	0.902	−0.164	0.186	3.34
Master or above	0.031	0.740	−0.150	0.211	1.61	0.026	0.772	−0.148	0.200	1.61	−0.036	0.663	−0.196	0.125	1.61
Fresh graduate or not (reference: No)					1.4					1.4					1.4
Yes	0.012	0.842	−0.104	0.128		−0.004	0.946	−0.116	0.108		−0.005	0.921	−0.108	0.098	
Region of Guangxi (reference: Eastern)															
Southern	0.113	0.102	−0.023	0.248	1.55	0.116	0.081	−0.014	0.246	1.55	0.126	0.040	0.006	0.246	1.55
Western	−0.237	0.002	−0.389	−0.085	1.72	−0.122	0.101	−0.268	0.024	1.72	−0.139	0.043	−0.273	−0.004	1.72
Northern	−0.161	0.019	−0.296	−0.027	1.62	−0.146	0.027	−0.275	−0.016	1.62	−0.137	0.024	−0.257	−0.018	1.62
Central	−0.069	0.266	−0.192	0.053	2.02	−0.063	0.292	−0.181	0.054	2.02	−0.113	0.041	−0.222	−0.005	2.02
Standardized training grade (reference: Grade 1)															
Grade 2	−0.005	0.944	−0.152	0.141	2.84	0.002	0.978	−0.139	0.143	2.84	−0.043	0.519	−0.173	0.087	2.84
Grade 3	−0.048	0.562	−0.212	0.115	3.18	−0.015	0.852	−0.172	0.142	3.18	−0.100	0.177	−0.245	0.045	3.18
Training identity (reference: integrated master student)															
Commissioned trainees from other institutions	0.168	0.160	−0.066	0.402	6.4	0.182	0.114	−0.044	0.408	6.4	0.100	0.347	−0.108	0.308	6.4
Social trainees	0.174	0.063	−0.009	0.357	5.25	0.161	0.074	−0.016	0.338	5.25	0.129	0.119	−0.033	0.292	5.25
Staff of the training base hospital	0.230	0.152	−0.085	0.544	1.44	0.176	0.255	−0.127	0.479	1.44	0.307	0.031	0.027	0.586	1.44
Licensed physician qualification exam (reference: passed)					3.22					3.22					3.22
Not passed	0.014	0.853	−0.133	0.161		0.034	0.639	−0.108	0.175		0.047	0.476	−0.083	0.178	
Training major category (reference: emergency and critical care medicine)															
Stomatology	−0.121	0.437	−0.425	0.184	2.2	−0.248	0.098	−0.541	0.045	2.2	0.026	0.853	−0.245	0.296	2.2
Obstetrics, gynecology and pediatrics	−0.038	0.775	−0.297	0.221	3.37	−0.091	0.475	−0.340	0.159	3.37	−0.072	0.540	−0.302	0.158	3.37
Medical technology	−0.148	0.246	−0.399	0.102	4.05	−0.204	0.097	−0.446	0.037	4.05	−0.012	0.917	−0.234	0.211	4.05
General practice	−0.126	0.366	−0.399	0.147	7.41	−0.215	0.110	−0.478	0.048	7.41	−0.059	0.632	−0.302	0.183	7.41
Internal medicine	−0.075	0.530	−0.310	0.160	6.83	−0.127	0.271	−0.354	0.099	6.83	−0.012	0.911	−0.221	0.197	6.83
Surgery	−0.044	0.711	−0.280	0.191	6.53	−0.113	0.327	−0.340	0.113	6.53	−0.065	0.539	−0.274	0.143	6.53
Constant	4.215	<0.001	3.384	5.046		4.411	<0.001	3.610	5.211		3.035	<0.001	2.298	3.773	
*N*	1,214					1,214					1,214				
*R^2^*	0.058					0.0438					0.0569				
Adjusted *R*^2^	0.039					0.0245					0.0378				
F-test (df)	*F*(24, 1,189) = 2.87					*F*(24, 1,189) = 2.26					*F*(24, 1,189) = 3.44				
*p*-value for *F*-test	<0.001					0.0005					<0.001				
Breusch-Pagan *χ*^2^	113.1					83.94					3.83				
Breusch-Pagan *p*-value	<0.001					<0.001					0.0504				
Shapiro–Wilk *W*	0.8608					0.8394					0.9601				
Shapiro–Wilk *p*-value	<0.001					<0.001					<0.001				

Separate multivariate linear regression models for knowledge, attitude, and practice scores. *β* = standardized regression coefficient; 95%CI = 95% confidence interval; *VIF* = variance inflation factor for multicollinearity diagnosis. Categorical variables take the group labeled “Reference” as the reference category. *VIF*>5 suggests moderate multicollinearity, and VIF > 7 indicates severe multicollinearity observed in several major categories. Breusch-Pagan test for heteroskedasticity and Shapiro–Wilk test for residual normality were conducted. The training base hospital staff subgroup has a small sample size (*n* = 29), and the corresponding regression estimate has limited robustness.

## 4 Discussion

This study shows that resident physicians in Guangxi have relatively high levels of proactive health knowledge and beliefs, but their behavioral levels are low. This finding is consistent with the results of studies on the current status of KAP regarding proactive health management and chronic disease management among community general practitioners (27, 28). Related studies also indicate that although healthcare workers possess a wealth of health knowledge, their health status is not optimistic due to heavy workloads, chronic stress, and irregular work schedules (29). This indicates that a significant gap still exists between knowledge acquisition and behavioral translation, necessitating further research to explore the underlying mechanisms linking the two and other factors that may influence behavioral translation. By systematically investigating the current status of knowledge, attitudes, and practices (KAP) regarding proactive health management among resident physicians in Guangxi, and analyzing differences in KAP and key influencing factors among resident physicians with different characteristics, this study aims to provide empirical evidence for optimizing proactive health management strategies for resident physicians, improving their physical and mental health, and enhancing their professional competence. It also serves as a reference for similar studies in other regions of China.

In terms of knowledge, the overall average score for proactive health knowledge among resident physicians in Guangxi was 4.32 ± 0.72, placing them in the upper-middle range. The item with the highest score was “Understanding the health risks of smoking and recognizing the importance of quitting” (93.66%). This is primarily because the health risks of smoking are widely recognized as common health knowledge, and as medical professionals, residents receive relevant health education during both their academic training and residency programs, resulting in a solid grasp of this foundational health knowledge; The item with the lowest score was “Knowing how to obtain health support through online platforms (such as websites, apps, and WeChat official accounts).” This is presumed to be related to the lack of digital health access channels among some residents (especially those with rural household registration or from underdeveloped regions) and the absence of targeted digital health knowledge training during their residency program. These findings align with the conclusions of a study by Liu et al. (30), which noted that healthcare professionals’ mastery of health knowledge is uneven: while basic health knowledge is widely disseminated, knowledge regarding digital health remains insufficient. Univariate analysis revealed significant differences (*p* < 0.05) in knowledge dimension scores among residents based on gender, household registration type, educational level, training identity, and region: Women scored higher than men, likely due to their social and family roles, which typically lead them to pay more attention to health information (such as health education, nutritional knowledge, and disease prevention); non-agricultural household registrants scored higher than those with agricultural household registration, likely due to the scarcity of health education resources in rural areas; those with a master’s degree or higher scored the highest, while professional master’s students scored the lowest, reflecting that higher educational levels correlate with stronger knowledge acquisition capabilities; residents at training hospitals scored the highest, while those in the integrated professional master’s program scored the lowest. This is because the former have greater access to practical health management resources, whereas the latter face dual pressures from academic studies and residency training, resulting in limited time for knowledge acquisition; the southern Guangxi region scored the highest, while the western Guangxi region scored the lowest, which is related to the uneven distribution of regional medical resources and disparities in health training opportunities (31).

Regarding the attitude dimension, the overall average score for resident physicians’ proactive health beliefs was 4.40 ± 0.69, indicating a generally positive attitude. The item with the highest score was “Believing that quitting smoking can effectively reduce health risks and improve quality of life” (94.40%). This is closely related to the professional competence of resident physicians; through their long-term exposure to clinical practice, they have a deep understanding of the harm caused by unhealthy lifestyle habits and place a high value on proactive health management. The item with the lowest score was “Believing that actively seeking health information and support through health platforms is helpful for health management.” This was primarily due to some residents’ busy clinical schedules, which left them with insufficient time to use health platforms, coupled with a lack of trust in digital health services. These findings align with the conclusions of Abed et al. (32), whose study, based on the Health Belief Model, found that healthcare professionals held positive overall beliefs regarding proactive health, but there were variations in their acceptance of new health service models. Univariate analysis revealed significant differences (*p* < 0.05) in belief dimension scores among residents stratified by gender, household registration type, training identity, and geographic region. Female residents achieved higher belief scores than male counterparts; this gender disparity should not be simplistically interpreted as an inherent higher priority placed on health management by women. Instead, it may stem from gendered occupational stress, intensified work–family conflict, and disproportionate time poverty confronting female residents, who bear overlapping clinical workloads and unpaid domestic care burdens, thereby raising their perceived necessity of proactive health management as a buffer against cumulative occupational strain (33). Non-agricultural household registrants obtained higher scores than agricultural household registrants, indicative of divergent health cognition shaped by urban–rural disparities in health literacy and access to health resources. Residents receiving standardized residency training at dedicated training hospitals yielded the highest scores, whereas those in integrated professional master’s training programs had the lowest scores, likely because trainees at specialized training hospitals accumulate abundant frontline experience in clinical health management, while integrated master’s students receive limited systematic exposure to proactive health concepts. Trainees based in southern Guangxi posted the highest belief scores, whereas those in western and northern Guangxi had the lowest scores, which aligns with regional gaps in health education outreach and uneven allocation of medical and preventive health resources across the province.

In terms of practical dimensions, the overall average score for resident physicians’ proactive health behaviors was 3.89 ± 0.64, which was significantly lower than that of the knowledge and belief dimensions, indicating a disconnect between knowledge, beliefs, and actions. The item with the highest score was “Following medical advice and actively undergoing standardized treatment after a health issue has been diagnosed” (88.63%), which reflects the professional instinct of resident physicians as medical professionals to consciously practice health behaviors related to clinical diagnosis and treatment; the item with the lowest score was “Engaging in high-intensity exercise (such as badminton or basketball) every week.” The core reason lies in the fact that resident physicians have busy clinical rotations, frequent night shifts, and heavy workloads, leaving them with insufficient time and energy to engage in regular exercise. Additionally, some physicians exhibit a cognitive bias of “prioritizing patient care over their own health.” These findings are consistent with the results of Frank et al. (33). Based on the physician competency framework, their study confirmed that sustained and heavy clinical workload greatly compresses the personal disposable time of medical staff, and long-term occupational pressure creates multiple practical barriers, ultimately leading to the generally low implementation level of health behaviors such as regular exercise, healthy work and rest routines, and balanced diets among medical workers (34). Univariate analysis showed that the scores of the health behavior dimension differed significantly among resident physicians grouped by gender, education level, training identity, and regional distribution (*p*<0.05). Male resident physicians scored higher than females, because males are more inclined to adopt simple and efficient health behaviors, while females suffer from poor health behavior compliance due to the dual pressure of family and work. In terms of education level, resident physicians with a bachelor’s degree or below had the highest scores, while on-campus professional master’s students had the lowest scores, which was attributed to the dual academic and clinical training burdens that limit their available time and energy. In terms of training identity, staff working at training base hospitals showed the highest practice scores, whereas integrated master students had the lowest scores. This discrepancy may be explained by the superior health management conditions available for hospital staff and the difficulty for postgraduate trainees to balance academic tasks and personal health management. Regionally, residents in southern Guangxi had the highest scores and those in western Guangxi had the lowest scores, which is closely related to regional differences in medical resource allocation and clinical work pressure. Notably, the subgroup of training base hospital staff included only 29 participants, accounting for merely 2.39% of the total sample. Owing to the extremely small cell size, the finding of the highest practice score in this subgroup exhibits limited statistical robustness, with low precision and high sensitivity to individual outliers and sampling bias. Therefore, this result should be interpreted with caution and cannot be overgeneralized. Further validation with a larger sample size is required to confirm the present finding.

The results of multiple linear regression analysis demonstrated that after controlling for confounding factors, gender, household registration type, residential region, and training identity were the key factors affecting the proactive health KAP levels of resident physicians in Guangxi (*p* < 0.05). In terms of gender, male residents exhibited significantly lower knowledge (*β* = −0.239, *p* < 0.001) and belief scores (*β* = −0.183, *p* < 0.001), yet higher behavior scores (*β* = 0.085, *p* < 0.05) relative to female counterparts, revealing divergent patterns of health cognition and behavioral implementation across genders. This gendered knowing-doing mismatch aligns with core tenets of the Health Belief Model and the Theory of Planned Behavior: female residents report stronger health beliefs driven by gendered occupational stress, intensified work–family conflict and disproportionate time poverty, yet encounter more prominent perceived barriers to consistent health practice, while male trainees face fewer domestic care burdens that partially mitigate structural constraints on behavioral execution. For household registration status, rural household registration predicted significantly lower knowledge (*β* = −0.124, *p* < 0.05) and belief scores (*β* = −0.126, *p* < 0.05) compared with non-rural registration, a gap largely shaped by urban–rural inequities in health literacy access and preventive health resource distribution. Geographically, residents stationed in western and northern Guangxi attained overall lower KAP composite scores than those in southern and eastern Guangxi, a disparity linked to uneven regional economic development, imbalanced medical workforce allocation, and variable public health education coverage. Regarding training identity, staff working at training base hospitals presented significantly higher practice scores than other subgroups (*β* = 0.307, *p* < 0.05, 95%CI: 0.027–0.586). This group can rely on superior medical and health management resources in affiliated hospitals, enabling them to more effectively transform health cognition into practical health behaviors. Under the Theory of Planned Behavior framework, trainees at dedicated training hospitals gain robust behavioral control via institutional health management resources to convert health awareness into routine practice. In contrast, integrated professional master’s students are exposed to multiple overlapping academic, clinical and research pressures, which elevate perceived environmental barriers and reduce self-efficacy for sustained health behaviors, creating a pronounced intention-behavior gap that cannot be explained by workload alone (35). Consistent with classic health psychology observations of the knowing-doing gap, high health knowledge and positive belief intentions do not reliably translate into tangible health practices unless structural barriers and limited behavioral control are adequately addressed (36). Of note, owing to the small sample size of the training base hospital staff subgroup (n = 29, 2.39% of total participants), the above regression estimate has a wide 95% CI and limited robustness. This subgroup result should be interpreted with caution, and further large-sample validation is needed.

Based on the findings of this study, and taking into account the current status of proactive health management among resident physicians in Guangxi and its key influencing factors, the following targeted recommendations for improvement are proposed: First, implement targeted training tailored to specific groups. For male residents, those with rural household registrations, graduate students in integrated professional master’s programs, and residents in western and northern Guangxi, prioritize training in areas of weakness, such as digital health literacy and health behavior implementation skills (37, 38). Adopt a blended online-offline model to enhance the accessibility of training; Second, optimize residency training management plans by rationally allocating clinical rotation tasks to reduce the professional workload of residents, particularly the academic and training pressures faced by integrated professional master’s degree students, ensuring they have sufficient time for self-health management; Third, improve resource allocation and policy support by leveraging the “Internet + Health” model to prioritize health resources for rural and underdeveloped areas, establishing remote health training platforms to narrow regional disparities; Fourth, strengthen health education and guidance to correct the cognitive bias among residents that prioritizes clinical diagnosis and treatment over personal health. Incorporate proactive health management into the residency evaluation system to increase their initiative in practicing healthy behaviors; Fifth, establish a health service platform to provide residents with convenient digital health support, exercise guidance, and other services, thereby effectively transforming proactive health management from “knowledge and attitude” into “practice,” and comprehensively improving residents’ physical and mental well-being as well as their professional competence (39, 40).

## 5 Limitations

This study has several limitations. First, the cross-sectional design cannot support causal inferences. This point is further emphasized given the regression analyses applied in this work, which only reflect correlational relationships rather than causality. Second, the generalizability of the findings may be limited; although the sampling strategy ensured good representativeness of the western coastal region of China, the results may not be fully applicable to other domestic regions with distinct socioeconomic backgrounds or international medical training systems. Third, this study adopted self-reported questionnaires to measure health behaviors, which may introduce social desirability bias. Fourth, contradictory descriptions of the scoring method may bring potential measurement bias. Finally, workload-related confounding factors (working hours, night shifts, training stress) were not incorporated into the analytical models, and these factors could affect respondents’ overall proactive health KAP levels.

## 6 Conclusion

This study indicates that the overall level of KAP regarding proactive health among resident physicians in Guangxi is appropriate, with physician gender, household registration status, region, and residency status being the primary influencing factors. However, there is still room for improvement in the practice of proactive health behaviors, which may affect physicians’ understanding of the concept of proactive health. Therefore, when promoting the proactive health model, efforts should focus on developing targeted intervention strategies, narrowing urban–rural and regional disparities through policy support and information technology, incorporating health management into residency training assessments, and designing specialized courses to enhance physicians’ self-health management capabilities, thereby creating favorable conditions for further promoting public health.

## Data Availability

The original contributions presented in the study are included in the article/supplementary material, further inquiries can be directed to the corresponding authors.
